# Lifetime analysis of *mdx* skeletal muscle reveals a progressive pathology that leads to myofiber loss

**DOI:** 10.1038/s41598-020-74192-9

**Published:** 2020-10-14

**Authors:** Ryan T. Massopust, Young il Lee, Anna L. Pritchard, Van-Khoa M. Nguyen, Dylan A. McCreedy, Wesley J. Thompson

**Affiliations:** 1 Texas A&M Institute for Neuroscience, College Station, USA; 2 Department of Biology, Texas A&M, College Station, USA; 3 Department of Pharmacology and Therapeutics, University of Florida College of Medicine, Gainesville, USA; 4 College of Medicine Myology Institute, University of Florida, Gainesville, USA

**Keywords:** Cell growth, Mechanisms of disease, Motor control, Motor neuron, Neuromuscular junction

## Abstract

The muscular dystrophy X-linked mouse (*mdx*) is the most commonly used preclinical model for Duchenne muscular dystrophy. Although disease progression in the mouse does not perfectly model the human disease, it shares many pathological features. Early characterizations of the model reported severe pathology through early adulthood followed by disease stabilization. As a result, research in the *mdx* mouse has largely focused on early adulthood. The overarching goal of this study is to improve the understanding of the *mdx* mouse model by tracking pathological features of the disease throughout life. We performed a thorough characterization of myofiber pathology in *mdx* mice from 2 weeks to 2 years of age. We report that individual *mdx* muscle fibers undergo progressive hypertrophy that continues through the lifespan. Despite massive hypertrophy on the myofiber level, we report no hypertrophy on the muscle level. These seemingly contradictory findings are explained by previously underappreciated myofiber loss in *mdx* mice. We conclude that due to myofiber loss, in combination with the progressive nature of other pathological features, aged *mdx* muscle tissue provides reliable benchmarks for disease progression that may be valuable in testing the efficacy of therapeutics for Duchenne muscular dystrophy.

## Introduction

Duchenne muscular dystrophy (DMD) is an X-linked degenerative muscle disease that affects roughly 1 in 3,500 males^1^. The disease is characterized by cycles of myofiber degeneration and regeneration that lead to an eventual repair failure and replacement of muscle with fibrotic and adipose tissue. The loss of muscle fibers leads to progressive weakness, loss of ambulation and premature death^2^.

A mouse model of DMD, the *mdx* mouse, was discovered in the early 1980s in a colony of C57BL/10 mice as the result of a spontaneous mutation causing histological and functional pathologies characteristic of DMD^3^. As in DMD, *mdx* mice lack dystrophin protein^4^. Initial characterizations dismissed this strain as a viable experimental model for human muscular dystrophy, reporting that the mice undergo a severe bout of muscle damage and repair between 3 and 12 weeks of age, followed by a functional and anatomical recovery^5–7^. Indeed, some features of DMD are poorly replicated in the *mdx* mouse. For example, *mdx* mice do not progressively lose muscle strength and therefore the ability to ambulate^8^. They have been reported to have a more robust regenerative response than DMD patients leading to effective replacement of damaged muscle tissue rather than deposition of fibrotic and adipose tissue^6,9,10^. Perhaps because of this vigorous regenerative response, analyses of transverse sections report that myofiber loss does not occur in the *mdx* mouse and in some cases *mdx* muscles contain more myofibers than age matched wild-type (WT) mice^6,11^. As a result, the general consensus was that the *mdx* mouse is a good model for studying myofiber regeneration but not for human muscular dystrophy.

The subsequent discovery that the *mdx* mouse and human DMD patients share a common mutated causative gene generated new interest in the *mdx* mouse as a model for DMD^4^. As a result of this interest, the *mdx* mouse has been bred to different backgrounds such as DBA2 and numerous models with different mutations have been created in an effort to better model DMD^12^. The severity of pathology varies widely among these different models^13^. Today, the *mdx* mouse is the most widely used preclinical model for DMD, but there is still much to learn about the degree to which the model mimics the human disease. This is especially true in old age, as most reports focus on the first 6 months of life. Many features of DMD also manifest in the *mdx* mouse. For example, central nuclei, muscle hypertrophy, myofiber branching, and shortened lifespans are features of both the human disease and the mouse model^3,14–17^.

Reports that track pathological features into aged time points are few and far between, and some features, such as central nuclei, are challenging to track within the dense and opaque environment of skeletal muscle tissue. Therefore, most analyses have utilized cross sections to track these pathological features. However, there are several disadvantages to this preparation. One such disadvantage was recently demonstrated for assessing myofiber number in muscle from *mdx* mice. The authors found an increase in myofiber number counted by cross section, suggesting hyperplasia, in the third compartment of the *mdx* extensor digitorum longus (EDL) muscle. Myofiber number correlated with an increase in myofiber branching measured in isolated myofibers, but did not coincide with an increase in the number of neuromuscular synapses^18^. Since there is only one neuromuscular synapse on each myofiber^19^, they concluded that the increase in myofiber number as assessed in cross sections was the result of myofiber branching rather than true hyperplasia.

There is a need to assess *mdx* pathology with new techniques in order to overcome historical limitations of histological analysis. In these experiments we extend the knowledge of *mdx* pathology in aged time points and build on the results of Faber et al. to analyze myofiber number from a new perspective. We tracked hallmark features of dystrophy on a cellular and whole muscle level by isolating individual muscle fibers and clearing whole muscles of WT and *mdx* mice from 2 weeks to 2 years of age in order to characterize the degree to which the model mimics the human disease. Identifying which aspects of *mdx* pathology mimic DMD is essential in determining which therapeutic targets show promise to translate from mouse to human. Additionally, a thorough characterization of aged *mdx* muscles will provide a pathological benchmark to assess disease progression and therefore, the efficacy of therapeutics.

We report that many features of *mdx* pathology, such as endplate fragmentation, number of central nuclei and myofiber branching increase in severity throughout the first year of life and persist through the lifespan. Individual *mdx* muscle fibers undergo progressive hypertrophy that continues through the lifespan, unlike the human disease. Despite massive hypertrophy on the myofiber level, we report no hypertrophy on the muscle level. While these findings initially seem contradictory, they are explained by previously underappreciated myofiber loss in *mdx* mice. We therefore conclude that the *mdx* mouse is an excellent model for therapeutic strategies intending to prevent myofiber turnover or loss, but a poor model for one aimed at inducing myofiber hypertrophy. Finally, the progressive nature of the *mdx* pathology makes aged tissue valuable for assessing the efficacy of therapeutics.

## Materials and methods

### Mouse strains

C57BL/10ScSn-*Dmd*^*mdx*^/J mice (*mdx,* RRID:IMSR_JAX:001801) and C57BL/10ScSnJ (BL/10J*,* RRID:IMSR_JAX:000476) mice were purchased from Jackson Laboratory and bred in house. Use of these mice has been previously reported and all mice are available from Jackson Laboratory. C57BL/10ScSn-*Dmd*^*mdx*^/J were regularly bred against the C57BL/10ScSnJ background to minimize genetic drift. Three male mice per genotype (2) and age (8) were used for each experiment, totaling 48 mice for the experiments performed. All experimental procedures were approved by the Texas A&M University Institutional Animal Care and Use Committee (2016-0158 or 2019-0179) and in full compliance with the NIH Guidelines for the Humane Care and Use of Laboratory Animals.

### Single myofiber isolation

Mice were sacrificed via intraperitoneal injection of 0.15 mL of Euthosol (Med-Pharmex). Following sacrifice, myofibers were isolated as previously described^20^. Briefly, the hindlimb of the mouse was skinned and EDL muscles were dissected in a calcium chelating solution (137 mM NaCl, 5.4 mM KCl, 5 mM MgCl2, 4 mM EGTA, 5 mM HEPES, pH 7.0) to prevent contraction. Immediately after dissection muscles were pinned in Sylgard-lined dishes and stained with α-bungarotoxin (BTX) (1 mg/ml) conjugated to Alexa Fluor™ 555 (Thermo Fisher Scientific Cat# B35451) diluted 1:500 in calcium chelating solution for 2 h to label AChRs. After staining, the muscles were washed in calcium chelating solution 3 times for 5 min per wash. The muscles were then fixed in calcium chelated 4% paraformaldehyde (PFA) for 48 h at room temperature. After fixation, the muscles were washed in calcium chelating solution 3 times for 5 min per wash. Muscles were then bathed in 40% NaOH for 3 h at room temperature to degrade connective tissue. DAPI (1 mg/ml) (Thermo Fisher Scientific Cat# D3571) diluted 1:2000 in phosphate buffered saline (PBS) was applied to label nuclei for 20 min. Muscles were gently shaken for 8 min in PBS on a rocking platform. Forceps and a dissecting microscope were used to mount individual muscle fibers onto slides in anti-fade fluorescence mounting medium.

### Cross section analysis

Mice were sacrificed via intraperitoneal injection of 0.15 mL of Euthosol (Med-Pharmex). Following sacrifice, the sternomastoid (STM), soleus (SOL), and EDL muscles were dissected, weighed, and pinned at resting length in a Sylgard-lined dish and fixed using 4% PFA for 30 min. After fixation, the muscles were washed 3 times in PBS. Muscles were placed into a 30% sucrose solution overnight in preparation for freezing. Muscles were frozen in optimal cutting temperature blocks by exposure to isopentane cooled to − 80° C in liquid nitrogen. Muscles were sectioned through the belly of the muscle at a thickness of 15 μm. Sections were mounted on slides in preparation for staining.

Sections were labeled with wheat-germ agglutinin (1 mg/mL) conjugated to Alexa Fluor™ 488 (Thermo Fisher Scientific Cat# W11261) diluted 1:500, α-bungarotoxin (1 mg/ml) conjugated to Alexa Fluor™ 555 (Thermo Fisher Scientific Cat# B35451) diluted 1:500, and DAPI (1 mg/ml) (Thermo Fisher Scientific Cat# D3571) diluted 1:2000 in standard blocking solution. Sections were then washed 3 times with PBS and slides were coverslipped in anti-fade fluorescence mounting medium.

For picro-sirius red staining, sections were fixed in 4% paraformaldehyde for 15 min, washed in dH_2_0, then incubated in 0.2% phosphomolybdic acid (Polysciences Inc.) for 2 min. Sections were rinsed twice in dH_2_0, stained in 0.1% (w/v) Sirus red (Polysciences Inc) in picric acid (1.3%; Sigma) for 15 min, then washed twice in 0.5% (v/v) acetic acid (Sigma) in water. To prepare for mounting, sections were washed in water then dehydrated in a series of ethanol steps before clearing in Citrisolv (Decon Labs) for 5 min. Coverslips were then mounted using Cytoseal (Thermo Fisher).

For Oil-Red-O staining, sections were fixed in 4% paraformaldehyde (Electron Microscopy Services) for 15 min, washed in dH_2_0, then labeled with Oil-Red-O solution (3.75 g/L in 60% (v/v) isopropyl alcohol; Sigma-Aldrich) for 10 min. Sections were washed in dH_2_0 for 30 min and coverslips were mounted with 20 mM TRIS buffer (pH 8) in 50% (v/v) glycerol (Sigma-Aldrich).

### Muscle clearing

The following tissue clearing protocol was adapted from the MYOCLEAR protocol^21^. Mice were sacrificed via intraperitoneal injection of 0.15 mL of Euthosol (Med-Pharmex). Following sacrifice, we performed transcardial perfusion with ice cold 4% PFA. STM, EDL, and SOL muscles were dissected, weighed, and pinned at resting length in a Sylgard-lined dish and fixed overnight in 4% PFA at 4° C. Muscles were then washed in PBS overnight with 4 solution changes in preparation for the hydrogel/polymerization step.

Muscles were incubated in A4P0 (4% acrylamide, 0% paraformaldehyde) hydrogel solution at 4° C with gentle rocking overnight. The next day, samples were placed in a vacuum desiccator for 30 min. The chamber was then flooded with nitrogen gas and the tubes were capped and sealed. Muscles were incubated at 37° C for 3 h. Muscles were washed in PBS overnight with 4 solution changes in preparation for labeling.

Muscles were stained with α-bungarotoxin (1 mg/mL) conjugated to Alexa Fluor™ 488 (Thermo Fisher Scientific Cat# B13422) diluted 1:500 and Draq5 (Thermo Fisher Scientific Cat# 62,251) diluted 1:1000 in PBS with 0.01% sodium azide. Muscles were labeled at room temperature for 4 days, refreshing solution halfway through. Immediately after refreshing the staining solution, muscles were centrifuged for 2 h at 600 g in order to increase the depth of antibody penetration^22^. Subsequently, muscles were washed in PBS with 4 buffer changes over 24 h. After the third buffer change muscles were again centrifuged for 2 h at 600 g. Muscles were mounted in a column of agarose in a syringe with the tip removed. After allowing the agarose to solidify for 60 min, the syringes were placed in 10 mL of refractive index matching solution^18^. The syringe was then partially depressed lowering the column of agarose and the sample into the refractive index matching solution. The samples were rocked for 2 days protected from light to create a uniform refractive index throughout the sample.

### Image acquisition and analysis

Isolated myofibers were imaged with a Leica TPS II SP5 or Zeiss LSM 780 confocal microscope. Confocal image stacks were collected throughout the entire volume of each fiber using a 20 × (NA 0.4–0.8) objective with a 1.5 µm z-step size. The AChRs and synaptic nuclei were imaged with a 40 × (NA 1.4) oil immersion objective with a 0.3 µm z-step size. ImageJ was used for all measurements. Adobe Photoshop was used to stitch the images of entire myofibers from overlapping maximum projections of the image stacks. Myofiber length and diameter were measured using the ImageJ segment tool. Measurements of the diameter were taken in the XY and Z direction at 3 equidistant points along the length of the muscle and averaged. Volume was calculated by using the formula $\mathrm{V}=\uppi {\mathrm{r}}^{2}\times \mathrm{ l}$. Nuclear number, number of central nuclei, and number of branches were quantified from confocal stacks. Myonuclear domain was calculated by dividing myofiber volume by total number of nuclei. Junctions were considered fragmented if they had 5 or more separate receptor rich areas upon visual inspection. Fragmentation analysis was done using the threshold function and then the automated count function in ImageJ. The minimum object area was set to 5 μm^2^. Synaptic nuclei were counted by hand and categorized as synaptic if they met the following 2 criteria: they were within 1 μm of the sarcolemma and they were directly beneath the AChRs.

Cross sections were analyzed using a Leica DMRX epifluorescence microscope with a 20 × (NA 0.6) or 10 × (NA 0.3) objective. Cross-sectional area (CSA) was measured using the 20 × images with wheat germ agglutinin to outline myofibers. BTX staining was used to ensure that the section was taken within the endplate band in the center of the muscle. CSA was measured using the Myosoft plugin for ImageJ^23^. Three sections were analyzed per animal. Myofiber number was counted by hand using wheat germ agglutinin labeling in 10 × images of whole cross sections reconstructed from overlapping images. Picro-sirius red and Oil-Red-O stained sections were imaged using a Leica DM 6B upright microscope with a Leica DM4500 color camera. All images were captured at 5x (0.15 NA) magnification using LASX software. Three sections per animal were analyzed using the threshold function in ImageJ to analyze the area of the staining.

Cleared muscles were imaged using a Zeiss Z.1 light sheet microscope using a 5x (0.16 NA) objective. Whole muscles were imaged in 3–8 overlapping stacks so that entire muscles could be reconstructed. The 488 nm and 638 nm lasers were used to image the BTX and DRAQ5 staining respectively. Image stacks were imported in ImageJ and synapses were counted by hand through analysis of Z stacks.

### Statistical analysis

For analysis of picro-sirius red and Oil-Red-O staining, unpaired T-tests were performed. For all other experiments two-way ANOVA with Tukey post hoc analysis was used to assess significance between genotype and age. All statistical analyses were performed using Prism GraphPad 7 software. Statistical significance was set at P < 0.05.

For analysis of single fibers, 10 myofibers from each of 36 mice were analyzed for a total of 360 myofibers analyzed. Mice were grouped by age (2, 6, 12, 24, 52, 104 weeks) and genotype (*mdx* or C57BL10J). For analysis of whole muscles, mice were grouped by age (adult vs aged) and genotype (WT and *mdx*). N = 3 per group and one of each muscle analyzed (STM, SOL, EDL) was collected from each mouse. For analysis of mouse weight, N = 4 mice per group. For analysis of muscle weight, N = 3 mice per group and 6 muscles per group were weighed. For analysis of CSA, myofiber number in cross sections, synapse number in cleared muscles and analysis of picro-sirius red/Oil-Red-O, N = 3 mice per group were analyzed.

## Results

### *Mdx* mice undergo progressive myofiber hypertrophy and hypernucleation

**Figure 1:**
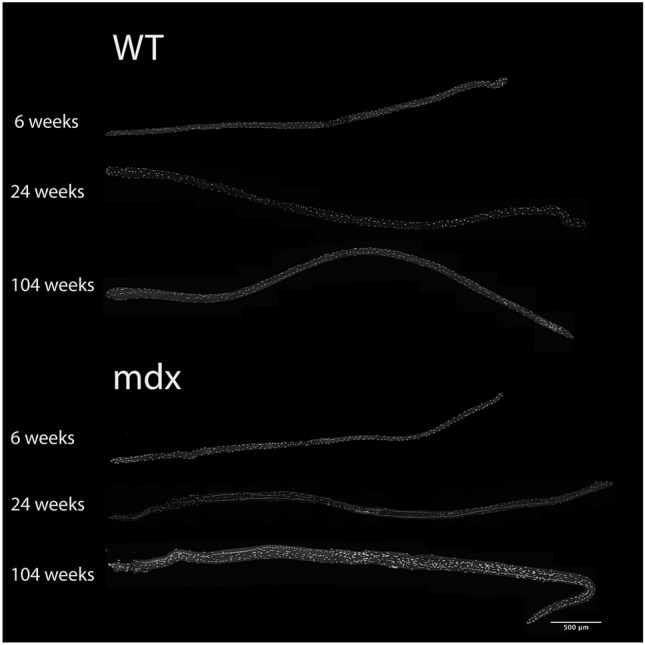
Representative images of myofibers from WT and *mdx* EDL muscles at various ages throughout the lifespan. Myofibers are stained with DAPI to visualize nuclei. Scale bar = 500 μm.

**Figure 2:**
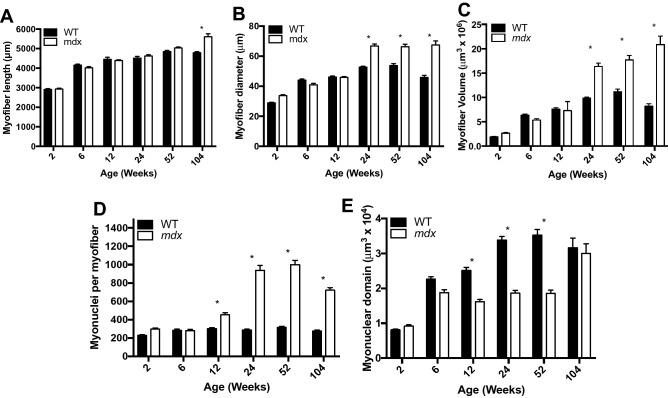
*Mdx* EDL myofibers are hypertrophic, hypernucleated and have a higher density of myonuclei relative to WT. (**A**) The length of *mdx* myofibers is not different from WT mice until 2 years of age, at which time extensive branching architecture occurs in *mdx* muscles. (**B**) The diameter of *mdx* myofibers is larger than age matched WT starting at 24 weeks and continuing through the lifespan. (**C**) *Mdx* myofibers become progressively hypertrophic throughout the lifespan and are not susceptible to atrophy in old age. (**D**) *Mdx* myofibers become hypernucleated by 12 weeks of age demonstrating recruitment of new myonuclei prior to the onset of hypertrophy. Hypernucleation increases through the first year of life and remains elevated through the lifespan. (**E**) The myonuclear domain of *mdx* mice is smaller than WT from 12 to 52 weeks of age indicating a higher density of myonuclei. N = 3 mice and 30 myofibers per group. Analysis via two-way ANOVA with Tukey post hoc test. * = p < 0.05. All data sets are displayed as mean ± s.e.m.

One of the signature features of DMD is muscle hypertrophy that occurs in the early stage of the disease, followed by muscle atrophy as the disease advances^17,24^. In order to determine if *mdx* myofibers follow the same pattern of growth, we isolated single myofibers from the EDL (Fig. 1) and tracked three aspects of myofiber size—length, diameter, and volume—throughout the lifespan. Myofiber length develops normally in *mdx* mice, showing no difference from WT through the first year of life. However, at 2 years of age *mdx* myofibers are longer than WT, likely due to complex branching architecture seen at high rates in aged *mdx* mice (Fig. 2A). Myofiber diameter also develops normally in *mdx* mice through the first 12 weeks of life, however, by 24 weeks, *mdx* myofibers have significantly larger diameters than WT and this increase persists through the lifespan (Fig. 2B). Myofiber volume develops normally in *mdx* mice through the first 12 weeks of life, but beyond that myofibers become progressively hypertrophic (Fig. 2C). By 2 years of age *mdx* myofibers are 155% larger than age-matched WT myofibers (Fig. 2C). Interestingly, *mdx* myofibers do not seem to be susceptible to atrophy associated with sarcopenia. These results indicate that the myofiber hypertrophy seen in *mdx* is progressive and does not follow the pattern seen in DMD. Previous reports demonstrate that hypertrophy induced in WT muscles is preceded by an increase in myonuclei^25^. This is thought to be necessary to support the increased volume of the cell.

We tracked the number of myonuclei in myofibers throughout the lifespan of *mdx* and WT mice. The number of nuclei in *mdx* myofibers is no different from WT for the first 6 weeks; however, starting between 6 and 12 weeks *mdx* myofibers become hypernucleated (Fig. 2D). This hypernucleation increases throughout the first year of life and then decreases between 1 and 2 years of age. The increase in myonuclear number precedes myofiber hypertrophy as expected from previous reports on WT myofiber hypertrophy^25^.

The relationship between myofiber volume and myonuclear number is accounted for in a statistic called myonuclear domain, which is the average volume of sarcoplasm per myonucleus^26^. Dysregulation of the myonuclear domain is a pathological feature of human muscular dystrophies as well as spinal muscular atrophy^27,28^. In WT mouse myofibers the myonuclear domain increases steadily through the first year of life as a result of slow growth of the myofiber without the addition of myonuclei. Between 1 and 2 years of age, WT myonuclear domain decreases due to a loss of myofiber volume without a loss of myonuclei. This is in stark contrast to what we observe in *mdx* myofibers where there is growth of the myonuclear domain between 2 and 6 weeks followed by a preservation of the myonuclear domain between 6 weeks and 1 year of age (Fig. 2E). The *mdx* myonuclear domain is significantly smaller than WT at 12, 24, and 52 weeks indicating that there is a higher density of myonuclei in *mdx* myofibers at these ages. The relationship breaks down in old age when *mdx* fibers continue to grow in size but lose myonuclei. The preservation of myonuclear domain size in *mdx* mice despite ongoing muscle fiber damage and repair indicates that processes within the myofiber regulate the myonuclear density. It is possible that the reduced myonuclear domain size in *mdx* mice corresponds to the increased protein demands of a cell tasked with repeated repair.

### Myofiber branching and central nuclei contribute to hypertrophy and hypernucleation respectively

**Figure 3:**
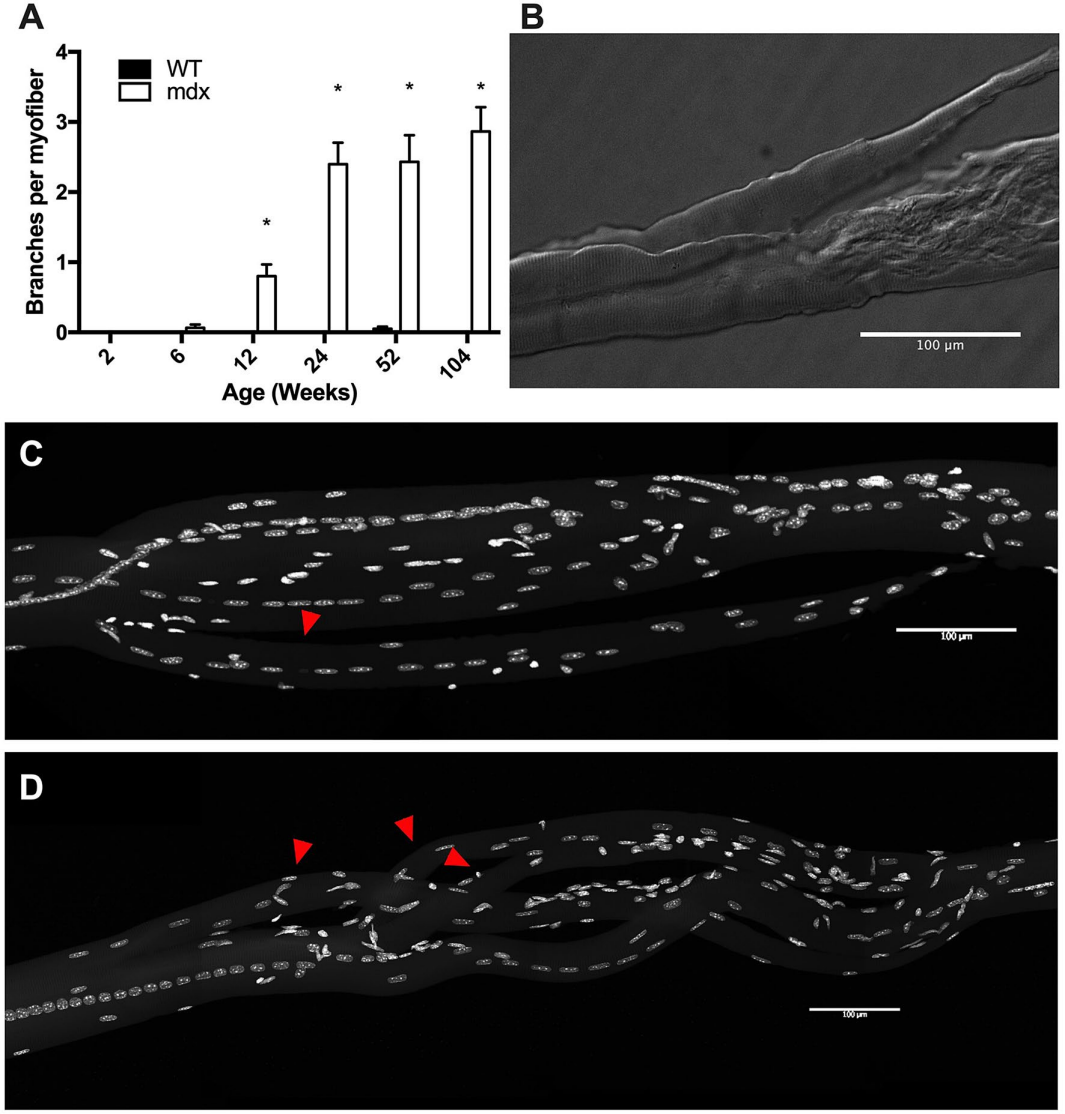
*Mdx* EDL myofibers have complex branching architecture. (**A**) Myofiber branching increases with age in *mdx* mice. No significant branches were observed in WT mice. (**B**) Differential interference contrast image of a damaged myofiber with a branch from a 24-week-old *mdx* mouse. (**C**) An example of a myofiber branch from a 52-week-old *mdx* mouse. Red arrowhead identifies the branch. Nuclei are stained with DAPI. (**D**) An example of complex branching architecture seen in 104-week-old *mdx* mice. Red arrowheads identify branches. Nuclei are stained with DAPI. Scale bars = 100 μm. N = 3 mice and 30 myofibers per group. Analysis via two-way ANOVA with Tukey post hoc test. * = p < 0.05. All data sets are displayed as mean ± s.e.m.

Branched myofibers have been shown to have a larger diameter than their non-branched counterparts^18^. While no significant branching occurs in WT mice, we found that branching started in *mdx* EDL muscles between 6 and 12 weeks of age and increased in severity throughout the lifespan such that by 2 years of age the average *mdx* myofiber contained approximately 3 separate branches (Fig. 3A). Branching architecture in these myofibers is complex and irregular, often branching several times in close proximity or rerouting the fiber in direction that is not parallel to the length of the muscle itself (Fig. 3B–D). These branch points have previously been demonstrated to be sites of mechanical weakness and as such are likely to contribute to cycles of myofiber damage^15,29,30^. We observed that myofiber branches rarely feature synapses. In our analysis of over 300 *mdx* myofiber branches, we found only a single instance of branching with an acetylcholine receptor (AChR) aggregate. In this instance, the branch occurred directly through the junctional area and the two aggregates lined up next to each other on their respective branches.

**Figure 4:**
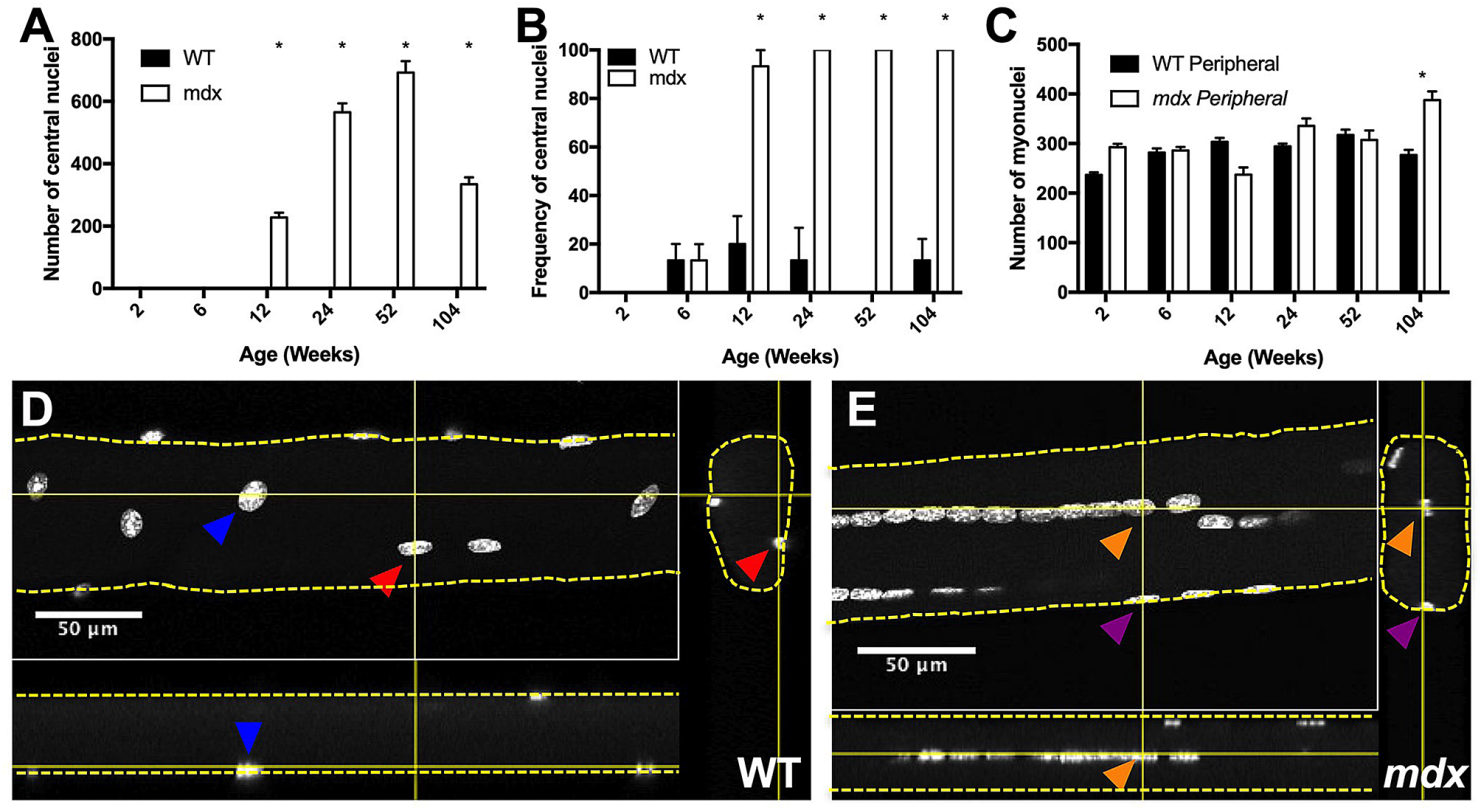
*Mdx* EDL myofibers have a high number and frequency of central nuclei relative to WT. (**A**) *Mdx* myofibers accrue progressively more central nuclei throughout the first year of life. Central nuclei number decreases in the second year of life, coincident with a decrease in total myonuclear number. (**B**) Frequency of myofibers containing central nuclei at any point along their length. By 24 weeks of age 100% of *mdx* myofibers contain central nuclei. (**C**) *Mdx* and WT myofibers have same number of peripheral nuclei through the first year of life. This indicates that central nuclei account for hypernucleation in *mdx*. (**D**) Confocal image of a 24-week-old WT myofiber containing peripheral nuclei with orthogonal views. Solid yellow lines indicate virtual slices. Dashed yellow lines outline myofibers. Color coated arrowheads identify the same myonucleus from different views. (**E**) Confocal image of a 24-week-old *mdx* myofiber containing peripheral and central nuclei with orthogonal views. Solid yellow lines indicate virtual slices. Dashed yellow lines outline myofibers. Orange arrowheads identify a central nucleus from different views. Purple arrowheads identify a peripheral nucleus from different views. Scale bars = 50 μm. N = 3 mice and 30 myofibers per group. Analysis via two-way ANOVA with Tukey post hoc test. * = p < 0.05. All data sets are displayed as mean ± s.e.m.

The presence of central nuclei in myofibers is a marker of myofiber repair and has long been recognized as a sign of diseased muscle tissue^31,32^. Mixed peripheral and central nuclei are common in *mdx* muscle fibers^33^. Here, we categorized each myonucleus as central or peripheral based on its location relative to the sarcolemma. WT myofibers do not have significant numbers of central nuclei at any time point. However, *mdx* myofibers begin to accrue large numbers of central nuclei between 6 and 12 weeks, indicating myofiber repair has taken place. The number of central nuclei per myofiber increases through the first year of life, and then decreases between 1 and 2 years of age, following the same pattern as total myonuclear number (Fig. 4A). By 24 weeks of age, all myofibers analyzed contained central nuclei at some point along their length, indicating that 100% of myofibers underwent damage and repair in the first 24 weeks of life (Fig. 4B).

We assessed the number of peripheral and central nuclei as separate populations and observed that the number of peripheral myonuclei in *mdx* myofibers is not different from the total number of myonuclei in WT myofibers until 2 years of age (Fig. 4C). Therefore, the hypernucleation seen in *mdx* myofibers throughout the first year of life is completely accounted for by the presence of central nuclei. This replicates the finding first reported by the Partridge Lab^34^. Examples of central and peripheral nuclei are shown in Fig. 4D–E.

### *Mdx* mice undergo progressive endplate fragmentation and accumulation of synaptic nuclei

**Figure 5:**
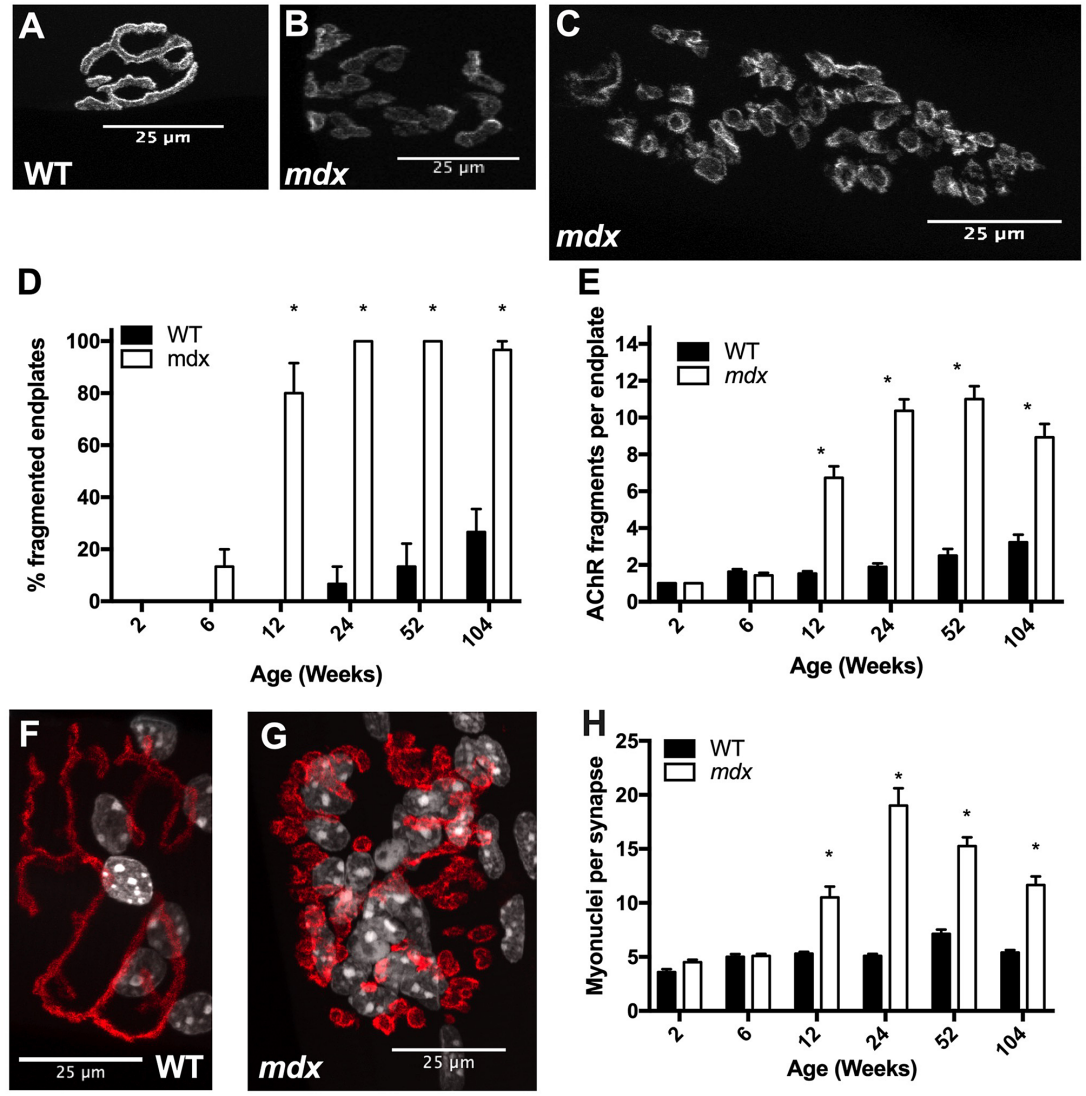
*Mdx* EDL myofibers undergo progressive endplate fragmentation and accumulate high numbers of synaptic nuclei. (**A**) Endplate from a 12-week-old WT mouse stained with BTX demonstrating the continuous “pretzel” like morphology. (**B**) Endplate from a 12-week-old *mdx* mouse stained with BTX demonstrating the fragmented morphology. (**C**) Endplate from a 52-week-old *mdx* mouse stained with BTX demonstrating the increase in severity of fragmentation with age. (**D**) Rates of endplate fragmentation are elevated in *mdx* mice. By 24 weeks of age, every endplate analyzed was fragmented. (**E**) The number of AChR fragments per endplate increases progressively through the first year of life. (**F**) 24-week-old WT endplate and synaptic nuclei stained with BTX (red) and DAPI (white). (**G**) 24-week-old *mdx* endplate and synaptic nuclei stained with BTX (red) and DAPI (white). (**H**) *Mdx* NMJs have higher numbers of synaptic nuclei starting at 12 weeks of age and continuing through the lifespan. Scale bars = 25 μm. N = 3 mice and 30 myofibers per group. Analysis via two-way ANOVA with Tukey post hoc test. * = p < 0.05. All data sets are displayed as mean ± s.e.m.

Fragmentation of the neuromuscular junction (NMJ) may have important implications for synaptic transmission. Fragmentation occurs in aging as well as in numerous disease states and has been induced by deliberate damage to muscles^35–38^ (Fig. 5A–C). In WT mice, endplate fragmentation was first observed between 12 and 24 weeks; by 24 weeks about 6% of endplates were fragmented. The frequency of fragmentation increased through the lifespan such that, by 2 years of age, roughly 26% of endplates were fragmented. In *mdx*, fragmentation first occurs between 2 and 6 weeks of age; by 6 weeks about 13% of endplates are fragmented. By 12 weeks the frequency of fragmented endplates jumps to 80% indicating that roughly 67% of myofibers underwent damage and repair to their NMJ between 6 and 12 weeks of age. At 24 weeks and beyond 100% of analyzed endplates were fragmented (Fig. 5D). In order to assess the severity of fragmentation, we quantified the number of discrete fragments per endplate. There was no difference between *mdx* and WT in the first 6 weeks of life. By 12 weeks of age *mdx* junctions have significantly more fragments than WT (Fig. 5E). Severity of fragmentation increases progressively in *mdx* mice through the first year of life but the number of fragments per endplate decreases between 1 and 2 years of age. This decline in old age may be due to receptor and/or fiber loss as well as denervation that occurs at this age. Regardless, the degree of NMJ fragmentation in *mdx* mice remains substantially greater than WT mice from 12 weeks of age onward.

Beneath each endplate, a subpopulation of myonuclei known as synaptic nuclei aggregate. These nuclei are tethered to the sarcolemma by Syne-1 and provide the transcripts necessary to maintain the AChR aggregate^39^ (Fig. 5F–G). We assessed the number of synaptic myonuclei beneath junctions to determine whether fragmentation of the AChR aggregate corresponds with changes in synaptic myonuclei populations. At WT neuromuscular junctions, there are approximately 5 synaptic myonuclei per junction at all time points assessed. However, in *mdx* mice, we observed a sharp increase in the number of synaptic myonuclei starting between 6 and 12 weeks of age. The number of synaptic myonuclei peaks at 24 weeks of age before steadily decreasing with age (Fig. 5H). Even with the decrease in old age, *mdx* mice have elevated numbers of synaptic myonuclei relative to WT at 12, 24, 52, and 104 weeks. These results are consistent with previous reports^40^.

### *Mdx* muscle weights are similar to wild type

**Figure 6:**
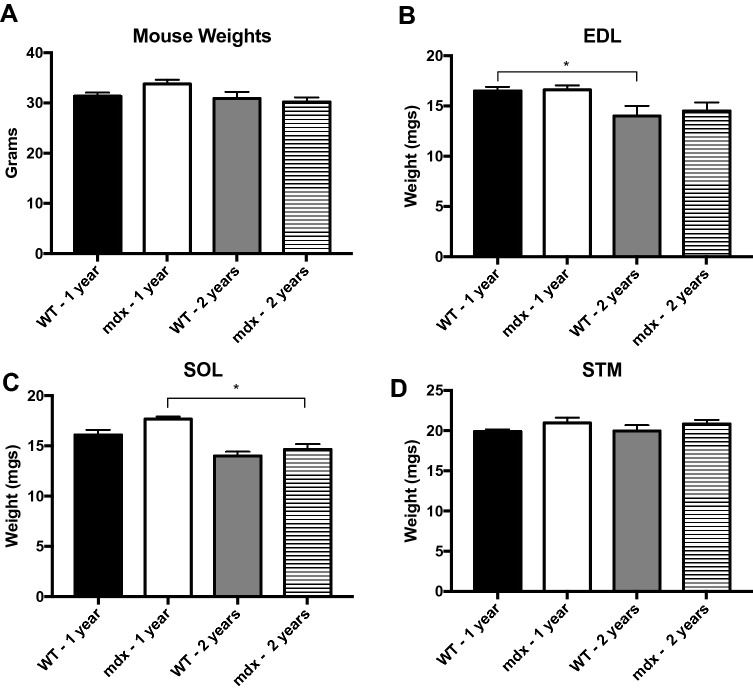
There is no difference in weight of age matched *mdx* and WT mice/muscles. (**A**) There are no significant differences in mouse weight across genotype or age. (**B**) WT EDL muscle weight significantly decreases between 1 and 2 years of age. There is a trend towards the same finding in *mdx*. (**C**) *Mdx* SOL muscle weight significantly decreases between 1 and 2 years of age. There is a trend towards the same in finding in WT. (**D**) There is no difference in STM muscle weight between any group. Analysis via two-way ANOVA with Tukey post hoc test. * = p < 0.05. All data sets are displayed as mean ± s.e.m.

Our analysis of isolated muscle fibers demonstrated progressive hypertrophy into old age; however, we did not notice a corresponding whole muscle hypertrophy. We observed no significant differences in body weights between age matched groups (Fig. 6A). Therefore, we recorded the wet weight of whole sternomastoid (STM), Soleus (SOL) and EDL muscles from WT and *mdx* mice during adulthood (11–14 months) and old age (21–24 months) to determine if myofiber hypertrophy contributes to whole muscle hypertrophy. There were no differences between age matched *mdx* and WT muscle weight in any of the three muscles (Fig. 6B–D). However, in the EDL we observed significant loss of muscle weight between adult and aged WT mice, and a trend towards the same loss in *mdx*. In the SOL there was a significant loss of muscle weight between adult and aged *mdx* mice, and a trend towards the same loss in WT. This trend was not observed in the STM muscle. This pattern of results fits well with previously published research on WT muscle size^41^ but appears to stand at odds with our current findings that individual muscle fibers are hypertrophic.

### Cross-sectional analysis shows no difference in cross-sectional area and myofiber number between age matched *mdx* and wild type muscles

**Figure 7:**
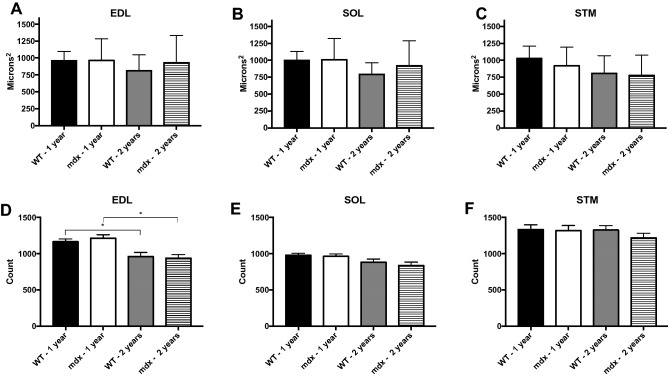
There are no significant differences in age matched CSA and myofiber number between *mdx* and WT. However, there is greater variability in the CSA of *mdx* muscles relative to WT. (**A**–**C**) CSA of adult and aged *mdx* and WT EDL, SOL and STM muscles. There are no significant differences between groups. (**D**) Myofiber numbers in WT and *mdx* EDL muscles. There are no differences in myofiber number between *mdx* and WT age matched groups; however, both WT and *mdx* EDL muscles lose a significant number of myofibers as they age. (**E**) Myofiber numbers in WT and *mdx* SOL muscles. There are no differences in myofiber number between any groups in the SOL muscle. (**F**) Myofiber numbers in WT and *mdx* STM muscles. There are no differences in myofiber number between any groups in the STM muscle. Analysis via two-way ANOVA with Tukey post hoc test. * = p < 0.05. All data sets are displayed as mean ± s.e.m.

In the previous section, we demonstrated that isolated myofibers are hypertrophic, in part due to their increased diameter. From these results, it would be logical to expect that cross-sectional area of *mdx* muscle fibers would also be larger. However, previous reports show no difference in mean cross-sectional area between *mdx* and WT, at least during adulthood^7^. However, *mdx* myofibers have increased variability in their cross-sectional area due to what has been described as two distinct populations of fibers^7,11^. First are recently regenerated myofibers with small cross-sectional areas; second are large hypertrophic myofibers. These populations together yield the same mean cross-sectional area as WT but introduce increased variability. Our findings are in line with previous reports and replicate well established findings using traditional analysis. In short, we find no difference in mean myofiber cross-sectional area between *mdx* and WT age matched groups (Fig. 7A–C).

We also replicated traditional myofiber number counts via cross section. Our findings align well with previous reports^11^; we observed no significant difference in myofiber number between age-matched groups (Fig. 7D–F). Previous research indicates that WT EDL and SOL muscles undergo significant myofiber loss associated with sarcopenia between adult and aged time points but the STM is resistant to this change^41^. We observed a significant loss of muscle fibers in the EDL muscle of WT and *mdx* mice between adult and aged populations. However, we observed no significant myofiber loss in the SOL or STM.

Previously, similar results have been interpreted to mean that *mdx* muscles do not undergo myofiber loss as seen in DMD^6^. However, in cross section preparations there is no reliable method to distinguish a myofiber branch from an independent myofiber. Given the significant degree of branching in adult and aged *mdx* muscles, there is evidence that substantial myofiber loss is being missed in this analysis due to counting myofiber branches as independent myofibers^15,18,41^. In order to assess myofiber loss without bias by branching we counted synapses in cleared whole skeletal muscles.

### Synapse counts in cleared tissue indicate significant myofiber loss in *mdx* mice

**Figure 8:**
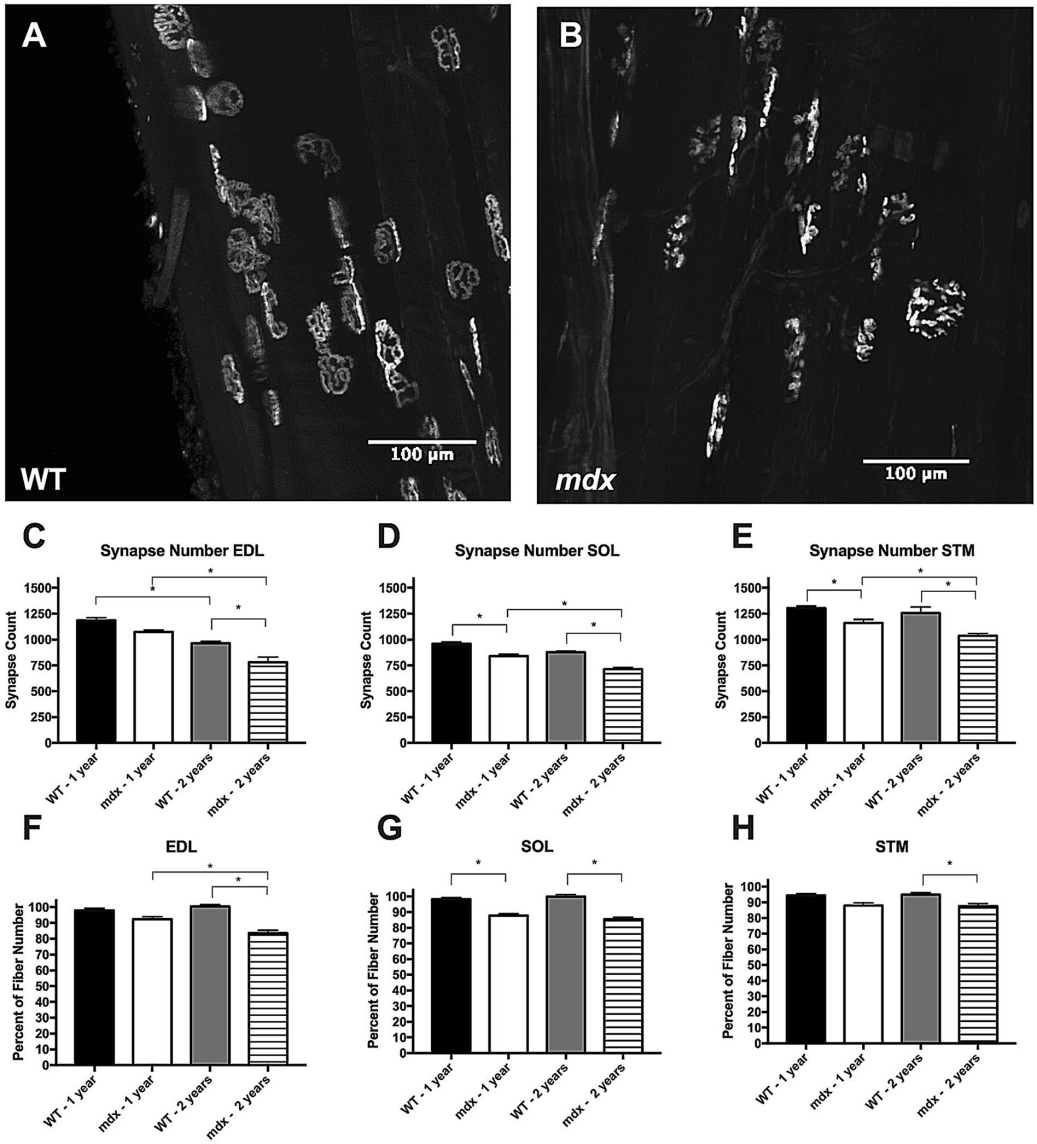
*Mdx* mice are susceptible to myofiber loss. (**A**) BTX labeled endplates from a cleared adult WT EDL muscle. Most endplates display the normal continuous morphology. (**B**) BTX labeled endplates from a cleared adult *mdx* EDL muscle. Most endplates display the fragmented morphology. (**C**) Synapse loss occurs as a function of age in WT and *mdx* EDL muscles. At 2 years of age, *mdx* EDL muscles have significantly fewer synapses than age matched WT. (**D**) Synapse loss occurs as a function of age in *mdx* SOL muscles and trends towards the same in WT. At 1 and 2 years of age *mdx* SOL muscles have significantly fewer synapses that age matched WT. (**E**) Synapse loss occurs as a function of age in *mdx* STM muscles. At 1 and 2 years of age *mdx* STM muscles have significantly fewer synapses than age matched WT. (**F**) Synapse number normalized against group matched myofiber number in the EDL muscle. The ratio of myofiber count to synapse count is significantly lower in aged *mdx* muscles compared to adult *mdx* and aged WT groups. (**G**) Synapse number normalized against group matched myofiber number in the SOL muscle. The ratio of myofiber count to synapse count is significantly lower in *mdx* muscles compared to age matched WT. (**H**) Synapse number normalized against group matched myofiber number in the STM muscle. The ratio of myofiber count to synapse count is significantly lower in aged *mdx* muscles compared aged WT muscles. Analysis via two-way ANOVA with Tukey post hoc test. * = p < 0.05. All data sets are displayed as mean ± s.e.m.

There is a robust one-to-one relationship between muscle fibers and neuromuscular synapses^19^. We analyzed over 300 branches for synapses and only found a single instance of a branch containing its own synapse, indicating this-one to-one relationship is preserved even in the instance of severe myofiber branching associated with the *mdx* pathology. Here, we utilize this information to count synapse number in cleared muscle tissue as a proxy for myofiber number (Fig. 8A,B). This strategy is employed in order to avoid the bias induced by counting myofiber branches in cross sections.

WT mice undergo sarcopenia as a part of the normal aging process. This manifests itself both as a loss of muscle fibers and atrophy of remaining myofibers^43–46^. WT synapse counts followed the same pattern of results as WT myofiber counts. In WT EDL muscles, we observed a significant loss of synapses between adult and aged populations, but not in the SOL or STM. In *mdx*, all three muscles underwent significant synapse loss between adult and aged time points (Fig. 8C–E), a deficit not seen with counts of myofibers in muscle transverse sections.

At 1 year of age *mdx* SOL and STM muscles had significantly fewer synapses than age matched WT, indicating that myofiber loss had already taken hold. Synapse loss increased in severity with age; in every case aged *mdx* muscles had significantly fewer synapses than age matched WT muscles indicating significant myofiber loss in *mdx* beyond that associated with the sarcopenia (Fig. 8C–E). WT synapse counts were on average 2.35% lower than their corresponding fiber count in transverse sections. The agreement of WT myofiber count via cross section and synapse count in cleared tissue indicates that counting synapses in cleared tissue is a reliable way to count myofibers. In comparison, synapse counts from adult and aged *mdx* muscles are on average 10.59% and 14.51% lower than their corresponding fiber counts respectively. By normalizing synapse count to age and genotype matched myofiber counts via cross section, we demonstrate that aged *mdx* muscles have significantly fewer synapses than would be predicted by traditional myofiber counts (Fig. 8F–H). The uniformity of these results across three different muscles with different fiber type compositions and body positions indicates that this feature of the *mdx* pathology is widespread and preserved across muscles. Together these results indicate that *mdx* muscles undergo significant myofiber loss associated with disease pathology and replicate a hallmark feature of DMD.

**Figure 9:**
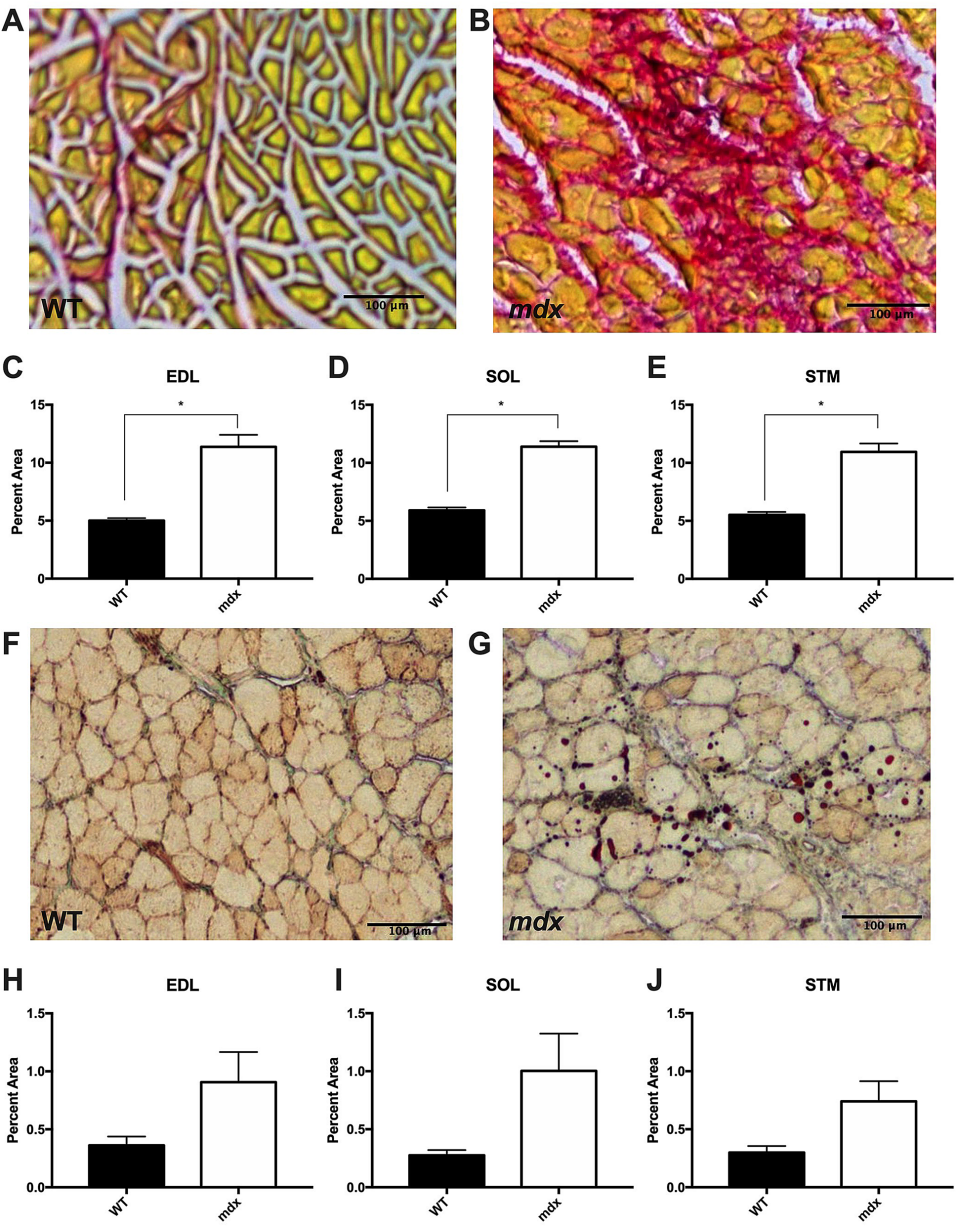
Aged *mdx* muscles contain more fibrotic tissue relative to WT. (**A**) Picro-sirius red staining for fibrotic tissue of aged WT EDL muscle. (**B**) Picro-sirius red staining for fibrotic tissue of aged *mdx* EDL muscle. (**C**–**E**) Aged *mdx* EDL, SOL and STM muscles contain significantly more fibrotic tissue than WT. (**F**) Oil-Red-O lipid staining of an aged WT STM muscle. (**G**) Oil-Red-O lipid staining of an aged *mdx* STM muscle (**H**–**J**) There is no significant difference in the lipid content of aged *mdx* and WT muscles, although there is a trend toward significance in the SOL and STM muscles (p < 0.1). Scale bars = 100 μm. Analysis via unpaired T-test. * = p < 0.05. All data sets are displayed as mean ± s.e.m.

The results presented thus far indicate that the size of *mdx* muscles are not different from WT despite loss of muscle fibers and hypertrophy of the remaining fibers. Previous research indicates that fibrosis in the *mdx* mouse is minimal in most muscles until aged time points^47^. Therefore, we tested the role that fibrosis and lipid deposition may play in the balance of muscle weight in aged *mdx* mice and found that aged *mdx* muscles have significantly more fibrotic tissue relative to WT, indicating that fibrosis also plays a role in compensating for loss of muscle fibers (Fig. 9A–E). However, we found no difference in lipid deposition in aged *mdx* muscles relative to WT (Fig. 9F–J), although there was a trend toward higher lipid content in *mdx* SOL and STM, but not EDL muscles.

## Discussion

These results demonstrate the progressive and persistent nature of the *mdx* pathology. By tracking pathological features through the lifespan of the *mdx* mouse, we identified that myofiber hypertrophy does not mimic the pattern of muscle growth and atrophy seen in DMD^48^. We performed whole muscle analysis to demonstrate that in three different muscles, aged *mdx* muscles have significantly fewer synapses than age matched WT muscles. Synapse number was used as a proxy for myofiber number to avoid bias by including non-innervated myofiber branches. This builds on a previous report^18^ and indicates that myofiber loss is a major feature of *mdx* pathology as it is in DMD^48^.

The hypertrophied calf muscle of DMD boys is perhaps the signature feature of early pathology; however, in humans the hypertrophy gives way to atrophy and regenerative failure^17^. In *mdx* mice, myofiber hypertrophy is progressive, such that in old age myofibers are at their largest. It is clear from these results that at least a subset of *mdx* myofibers are resistant to age-related atrophy that is seen in DMD and aging WT mice. It seems that a large contributor to this hypertrophy is myofiber branching, which increases both the diameter and to a lesser degree, the length, of myofibers. The persistent hypertrophy of *mdx* myofibers was unexpected and likely does not replicate the human disease. However, our analysis of whole muscle weight shows that at 1 and 2 years of age, *mdx* muscle weight is not different from WT. This finding is another report to be added to an issue that has been clouded by inconsistency. Reports on *mdx* muscle weight differ by muscle and age. Hypertrophy is reported during early adulthood (3–5 months) in *mdx* EDL and tibialis anterior (TA) muscles, but not in SOL^11^. Another report indicates the SOL and peroneus longus muscles are hypertrophic in 6 month old *mdx* muscles^49^. TA muscles from *mdx* mice are reported to be significantly smaller at 3–4 weeks of age but not different from WT during adulthood (2–12 months)^5^. Yet another report indicates that *mdx* TA muscles are hypertrophic through adulthood (2–12 months) but become atrophied beyond 15 months^10^. We report that aged *mdx* muscles are not atrophied, despite significant myofiber loss. This indicates that myofiber branching and hypertrophy, in combination with increased fibrosis, is responsible for the creation of enough new muscle volume to offset myofiber loss, but not enough to induce muscle hypertrophy.

In addition to myofiber hypertrophy we found that the severity of most pathological features in *mdx* mice tend to increase through the first year of life.

We have found that the frequency of central nuclei in isolated myofibers of *mdx* mice is over 93% at 12 weeks of age, indicating that 93% of myofibers have undergone repair of some scale by this age. This is higher than previous reports that rely on analysis of transverse muscle sections^9^, a discrepancy likely due to the fact that we analyzed isolated whole myofibers from tendon to tendon. Therefore, the result reported here is the frequency that central nuclei occur anywhere along the length of the myofiber, rather than within a given transverse section or series of sections. The number of central nuclei in *mdx* myofibers follows the same pattern as total myonuclear number, increasing progressively through the first year of life. The myonuclear domain of *mdx* mice is depressed throughout much of the first year of life, indicating a higher density of myonuclei. It is possible that the high rate of turnover in these diseased muscles requires a higher density of myonuclei in order to attempt to repair muscle more effectively. As *mdx* mice enter old age, they undergo a dramatic increase in myonuclear domain that is likely the result of continued myofiber hypertrophy coincident with myonuclear loss. It is easy to see how this could lead to myofiber damage and potentially loss as the decreased density of nuclei may not be able to support high demands of a cell in need of repeated repair. In addition, myonuclear movement may be compromised in *mdx* mice as a result of disorganized microtubule architecture^50^. The activity and effectiveness of myonuclei in *mdx* is an area deserving of further investigation.

Myofiber branching has been shown to be a result of the repair process from many different types of muscle injury^15,36,37,42^. The branch points are sites of mechanical weakness that are prone to contraction induced injury and may lead to repeated turnover and increased branching as a result. In isolated myofibers we observed an increase in myofiber branching throughout the lifespan. By old age, *mdx* fibers have an average of 3 branches per myofiber, compared to wild-type mice, which display negligible branching. These branches contribute to increased myofiber volume, primarily through increased diameter. We observed that myofiber branches do not feature synapses, and this may have negative consequences for the propagation of action potentials and force production in muscle fibers.

We found that endplate fragmentation is dramatically elevated throughout the lifespan of *mdx* mice and increases in severity through the first year of life. Previous reports have induced endplate fragmentation by injection of myotoxic drugs, physically damaging myofibers and by eccentric contraction protocols^37,51,52^. These results indicate that fragmentation can be the result of full myofiber replacement or from local damage and repair. In old age, *mdx* mice have slightly fewer AChR fragments per junction than at 1 year old. This result is unexpected. Although evidence exists that NMJs can revert to a less fragmented morphology in cases of exercise and caloric restriction^35^, there is little reason to anticipate that aged *mdx* mice would go through this process. There are several other possible explanations for this observation. First, aged *mdx* mice are undergoing partial or full denervation at their NMJs. As a result, some AChR fragments are no longer receiving axonal input and are lost. Indeed, some evidence exists that this is the case. When a partial block is put on a receptor aggregate using snake toxin, that area of the receptor is lost^53^. In addition, we observed some very faintly stained receptor fragments in aged *mdx* tissue that were excluded because they were under the intensity threshold required for analysis. These faint receptor fragments have been observed and reported previously^35,38^. Another possibility is that there is significant myofiber loss in aged *mdx* mice and that the loss preferentially occurs in a population of fibers that are more susceptible to damage. These same fibers, having undergone more cycles of degeneration and regeneration than their resistant counterparts, may also have a more severely fragmented endplate. This would leave behind a population of less severely fragmented endplates. We are aware of only one previous report that observes myofiber loss in *mdx*, but it is not quantified^54^.

In order to assess myofiber loss in *mdx* from a new perspective we utilized a variation of the MYOCLEAR technique to clear skeletal muscles and stain AChRs with fluorescent α-bungarotoxin^21^. This allowed us to assess the number of synapses in muscle tissue, extending the technique used by Faber et al. into whole muscles^18^. We found that the STM and SOL muscles already had fewer synapses than age matched WT at roughly one year of age, indicating that myofiber loss has already started in these muscles. Over the next year, the severity of synapse loss increases. Aged *mdx* EDL, SOL and STM muscles have significantly fewer synapses than age matched WT muscles. It is important to note that because we cannot measure myofiber number directly while accounting for myofiber branching, these results rely on synapse counts as a proxy for myofiber number. Therefore, we cannot rule out that there may be alterations in synapse number in *mdx* tissue that do not reflect myofiber loss. However, we believe the results presented here provide strong evidence that *mdx* mice are susceptible to myofiber loss and replicate an important feature of DMD that drives loss of function and quality of life in humans.

While the present study identifies loss of myofibers in *mdx* muscles, the factors that enable some fibers to be resistant to damage are still unclear. One possibility is upregulation of utrophin. Utrophin is structurally similar to dystrophin and overexpression of utrophin has been to demonstrated to reduce myofiber turnover in *mdx*^55–58^. In addition, upregulation of utrophin has been correlated with less severe disease progression in DMD patients^59^. It is possible that the remaining fibers in aged *mdx* muscle express higher relative levels of utrophin, decreasing the frequency of myofiber damage events. Another possibility is that different fiber types have different susceptibility to damage events. Indeed, the diaphragm muscle, primarily composed of fast twitch fibers, has been shown to be severely affected relative to other muscles^47^. In addition, the fast twitch EDL muscle is more susceptible to lengthening contractions and is preferentially affected in DMD^60–62^. It will be important for future work to identify characteristics of loss resistant myofibers.

In summary, we found that in *mdx* mice pathological features associated with cycles of myofiber damage and repair such as endplate fragmentation, deposition of central nuclei and myofiber branching all increase progressively through the first year of life. This is a strong indication that myofiber degeneration and regeneration is ongoing in the *mdx* EDL muscle. Myofiber hypertrophy in the *mdx* EDL continues progressively throughout the lifespan rather than becoming atrophied with age as in DMD, and does not correlate with whole muscle hypertrophy. Instead, the myofiber hypertrophy is offset by significant myofiber loss in *mdx* mice that occurs in three different muscles that vary in fiber type makeup and body position. We conclude that the pathology of *mdx* mice is progressive and that morphological changes in myofibers and at the NMJ of aged *mdx* mice are excellent benchmarks by which to assess disease progression.

## Data Availability

The datasets generated during and/or analyzed during the current study are available from the corresponding author on reasonable request.
